# *Plasmodium vivax* pre-erythrocytic vaccines

**DOI:** 10.1016/j.parint.2021.102411

**Published:** 2021-10

**Authors:** Arturo Reyes-Sandoval

**Affiliations:** a The Jenner Institute, University of Oxford, Old Road Campus Research Building, Roosevelt Drive, Oxford OX3 7DQ, UK; b Instituto Politécnico Nacional, IPN, Av. Luis Enrique Erro s/n, Unidad Adolfo López Mateos, Mexico City, Mexico

**Keywords:** *Plasmodium vivax*, Vaccine, Clinical trials, PEV, Pre-erythrocytic vaccines, BSV, Blood-stage vaccines, TBV, Transmission blocking vaccines

## Abstract

An estimated 229 million cases of malaria occurred worldwide in 2019. Both, *Plasmodium falciparum* and *P. vivax* are responsible for most of the malaria disease burden in the world. Despite difficulties in obtaining an accurate number, the global estimates of cases in 2019 are approximately 229 million of which 2.8% are due to *P. vivax*, and the total number of malaria deaths are approximately 409 million. Regional elimination or global eradication of malaria will be a difficult task, particularly for *P. vivax* due to the particular biological features related to the hypnozoite, leading to relapse. Countries that have shown successful episodes of a decrease in *P. falciparum* malaria, are left with remaining *P. vivax* malaria cases. This is caused by the mechanism that the parasite has evolved to remain dormant in the liver forming hypnozoites. Furthermore, while clinical trials of vaccines against *P. falciparum* are making fast progress, a very different picture is seen with *P. vivax*, where only few candidates are currently active in clinical trials. We discuss the challenge that represent the hypnozoite for *P. vivax* vaccine development, the potential of Controlled Human Malaria Challenges (CHMI) and the leading vaccine candidates assessed in clinical trials.

## 1 Introduction

*Plasmodium vivax* is the most prevalent human malaria parasite in the world and the one that presents most challenges in the route towards malaria elimination. An estimate in 2017 indicated that there were up to 3.3 billion people living within regions of *P. vivax* transmission [1]. The high disease burden is mainly due to its presence in the most densely populated regions, particularly in South East Asia with particular emphasis in India, but also in the Western Pacific Region.

Most scientific efforts towards impacting malaria elimination have historically focused on *P. falciparum*, despite the higher disease burden posed by *P. vivax*. This is partly due to the misconception of being a ‘benign’ form of malaria. Nevertheless, increasing evidence points towards an association with severe and fatal outcomes after *P. vivax* infection, with an apparent higher severe anaemia in areas of high transmission and resistance to chloroquine [2]. Various other studies in Indonesian Papua or India have challenged the perception of a ‘benign’ malaria and have attributed similar rates of mortality to both, *P. falciparum* and *P. vivax* when patients are hospitalized [3,4]. These studies show that approximately 21–27% of people with severe malaria corresponds to *P. vivax* [3,4]. Therefore, it is important to highlight that *P. vivax* malaria is far from a benign infection, and can lead to well-documented severe clinical features.

Global fund investment in the first decade of the 2000, was scarce for *P. vivax*, with 3.1% of the global funds dedicated to research and development. This is in contrast with 44.6% of malaria funds aiming to *P. falciparum* R&D [5]. This has resulted in a very low number of clinical trials specific for *P. vivax* in comparison with those to *P. falciparum*. (http://www.who.int/vaccine_research/links/Global_malaria_vaccine_portfolio.pdf) (www.ClinicalTrials.gov).

## 2 Towards a *P. vivax* malaria vaccine: roadblocks and challenges

Various major factors have been recognised as major challenges that have slowed down the progress towards *P. vivax* vaccine development [6]: high costs to develop new vaccines combined with lack of investment, scarcity of tools and logistic complications to perform vaccine efficacy studies in controlled human malaria infections (CHMI) [7]. CHMIs are perhaps a most useful feature of malaria trials, as substantial experience has increased in recent years and it has become a reality for *P. vivax* trials. Thus, CHMI becomes a necessary requirement before taking a vaccine candidate through the long and winding road of field trials, where costs grow exponentially. Nevertheless, CHMIs for *P. vivax* trials pose major logistic challenges that contribute to paucity in the vaccine development at the clinical trial stage. This is due to the lack of a continuous *in vitro* culture methodology to support *P. vivax* growth in research laboratories. Therefore, access to suitable *P. vivax* parasites is restricted to endemic countries where blood samples of infected volunteers are available. This has been possible mainly from Thailand [8] and within Colombia [9,10].

The cost associated with the development of a vaccine is also a major roadblock that may explain the slow pace to produce efficacious vivax vaccines. Studies estimate that the cost to produce a vaccine is between $600–800 million USD over a period of time of 10–15 years to take a vaccine to completion [11]. Drugs, for instance, can take half of the time and investment to be developed.

## 3 The hypnozoite

*P. vivax* has the ability to hide within hepatocytes for a variable time, weeks or years before reactivation. Upon activation, *P. vivax* will leave hepatocytes to infect erythrocytes, thus causing symptoms without any further sporozoite infection due to transmission by a mosquito bite. The latter clinical feature is known as relapse and it was first documented in 1897, where malaria symptoms appeared two years after an initial malaria episode [12]. Thus, *P. vivax* malaria parasites establish a latent reservoir, which explains the difficulty in eliminating the parasite from endemic regions using the traditional bednets and artemisinin-combination therapies (ACT), which have had a major impact in reducing the *P. falciparum* disease burden [13]. Therefore, targeting the hypnozoite and hence the relapse will certainly have to be at the front line of *P. vivax* malaria elimination, using hypnozoite-specific drugs and vaccines. For a vaccine to prevent relapse, an antibody response against the sporozoite could in theory prevent hypnozoite formation, while T-cells directed towards infected hepatocytes could potentially eliminate these cells to prevent relapse. Therefore, pre-erythrocytic vaccines are promising approaches in the plans for elimination and eradication of vivax malaria.

## 4 Controlled human malaria infections (CHMI)

Development of new vaccines with proven efficacy in humans is a lengthy process that can take years, in particular for neglected diseases such as *P. vivax*. Costs to complete a phase III efficacy trial are very high and the logistics to achieve this are also a major challenge. Both, time and costs can substantially be reduced when the pathogen can be used as a challenge model. This permits learning of the potential of a vaccine to protect against disease, supporting at the same time the investigation of protective correlates in a controlled environment. If available, CHMIs should be used before taking a vaccine on the longer and complex road of a phase III efficacy trial. CHMIs have been made possible for *P. vivax*, and trials have been reported for 5 pre-erythrocytic vaccines and 2 blood-stage approaches [7]. Unfortunately, *P. vivax* CHMIs are still in their early days of development and a major logistic challenge to perform such trials in a regular way, is the inability to grow *P. vivax in vitro*. For this reason, CHMIs require the use of fresh blood obtained from infected volunteers, as well as available mosquitoes to be infected. Unfortunately, few laboratories in the world are equipped for this.

## 5 The leading *P. vivax* vaccine candidates

It is remarkable that only very few *P. vivax* vaccines have been tested in clinical trials despite the significant disease burden caused by this parasite. Malaria vaccines are classified according to the stage of the life cycle targeted. They can be referred as pre-erythrocytic vaccines (PEV) when they aim to halt infection by the sporozoite recently introduced by a mosquito and before reaching the liver or before exciting this organ to start reproducing in red blood cells (RBCs). Blood stage vaccines (BSV) target the asexual stage of the parasite at the moment of reproduction in RBCs and transmission-blocking vaccines (TBV) prevent the sexual forms from developing within the mosquito.

Both, PEVs and perhaps to a less degree BSVs can benefit from humoral and cellular immune responses consisting of antibodies and cytotoxic T cells (CTLs) to target the parasite. Hence, when the parasite is at an extracellular environment, antibodies can block them or when in an intracellular compartment, CTLs can eliminate infected cells.

## 6 Pre-erythrocytic vaccines (PEVs)

PEVs are arguably the most attractive approach to prevent malaria and are still the leading vaccination strategy nowadays for *P. falciparum* [14]. PEVs are the only approach that have demonstrated sterile protection against malaria infection and this was achieved using radiation-attenuated sporozoites [15], a methodology still pursued nowadays as a vaccination strategy [16].

In addition, the pre-erythrocytic (PE) stage is considered a bottleneck where sporozoites reach only low numbers during infection, of approximately 10–100 hepatocytes are infected. In comparison, numbers of parasites to be neutralised during blood stages can reach up to 10^9^ or 10^11^ during sexual or asexual blood stages, respectively [17]. More importantly, no symptoms are present during the PE stage, thus, preventing the parasite from reaching the blood stage can also avoid severe symptoms or death.

The PE is considered a single stage. However, vaccines can induce an attack in two ways, in which immunity can target the sporozoites during the extracellular stage on their way towards the liver. Antibodies against sporozoite surface antigens play a major role to ‘neutralise’ the sporozoite before reaching the liver. Vaccine approaches like these would be known as anti-sporozoite vaccines. A second attack can be directed against the infected hepatocytes where CTLs can destroy cells that express parasite antigens through MHC class I molecules. These would be liver-stage vaccines. It is likely that an efficient approach should stimulate both, antibodies and CD8^+^ T cells against one or multiple antigens to the sporozoite and the infected hepatocyte stages. In this regard, approaches consisting of recombinant viral vectors, DNA or mRNA could be efficient ways to elicit such dual responses [[18], [19], [20]].

Plasmodium parasites have evolved a capacity to escape immune responses, particularly during blood stage. During the PE stage, free sporozoites are present in the blood for a brief period of time before reaching the liver. Perhaps the low number, coupled with a period of time during this extracellular phase, may make immune responses able to attack the parasite. Nevertheless, like many pathogens, surface antigens show variation in *P. vivax*, such as the circumsporozoite protein that has substantial amino acid variations that have led to their classification as VK210 or VK247 strains [21]. This should be taken into consideration for vaccine approaches to elicit immunity against multiple vivax strains.

## 7 Pre-erythrocytic *P. vivax* vaccines in clinical trials

The circumsporozoite protein (CSP) continues to lead the *P. vivax* field as a vaccine candidate. The CSP is the most abundant protein on the surface of mature sporozoites [22] and fulfils a vital role for the parasite by supporting gliding motility and invasion of hepatocytes [[23], [24], [25]]. In contrast to *P. falciparum*, a higher diversity is seen within the vivax CSP molecule and two major types are present around the world, VK210 and VK247, which differ in the central repetitive region of the molecule [21].

## 8 A synthetic peptide vaccine

Peptide synthesis can be relatively straightforward, becoming a suitable vaccine option. Current approaches for peptide synthesis permit the production of large peptides, with presence of disulfide bonds to support folding of the resulting molecule.

In addition, production can be fast and have good batch reproducibility and yet they can be produced under Good Laboratory Practices (GLP). Various vivax CSP long-synthetic peptides of approximately 70 amino acids in length have been synthesised, which comprise the N- and C-terminal regions, as well as the central VK210 repeat region [26].

Pre-clinical results in *Aotus* monkeys indicated that all animals developed antibodies that recognised the native CS protein on sporozoites, as well as peptide-specific T-cell responses using the ELISpot technique [27]. A clinical trial has been completed whereby malaria-naïve volunteers were immunised with the Long-synthetic Peptide (LSP) vaccine, in a dose escalating trial using 10 μg, 30 μg or 100 μg. Antibody responses were induced, in particular upon the administration of three doses of the highest vaccine dose [28].

## 9 The CSP-based Vivax-1 vaccine

This vaccine aims at inducing immune responses against the circumsporozoite (CSP) protein of *P. vivax* sporozoites. The immunogen is a recombinant protein that has 234 out of 373 amino acids of the CSP from the Belem isolate. Whilst it does not contain VK247 repeats, it has all the VK210 central repeat region, plus portions of the N- and C- terminal ends. The protein was produced in yeast, using *Saccharomyces cerevisiae* and then adsorbed onto Alhydrogel. Early pre-clinical results in mice yielded high antibody titers that also inhibited sporozoite invasion in hepatocytes [29]. Further pre-clinical assessment, using *Saimiri sciureus boliviensis* monkeys, had similar results with induction of high antibody titres elicited by the vaccine. The vaccine protected 67% of the animals and these results supported the vaccine transition towards clinical trials [30].

Results of this vaccine approach in clinical trials highlight how challenging vaccine development can be, as vaccines that show great promise in pre-clinical models may not have a similar outcome in humans. In a dose escalating phase I trial, safety and immunogenicity was assessed in a cohort of volunteers. The vaccine was prepared with Alhydrogel as adjuvant and administered to volunteers in doses of 50 μg, 100 μg, 200 μg, and 400 μg. Interestingly, despite 3 consecutive immunisations, none of the doses raised high antibody levels in humans, leading to the conclusion that a different formulation may be needed [31].

## 10 Vivax malaria protein 1 (VMP001) vaccine

*E. coli* can be a suitable option that supports production of large quantities of proteins under good manufacturing practices, suitable for vaccine use. This would permit reducing costs to have an affordable vaccine option. CSP has been produced in *E. coli* as a chimeric protein with the advantage of comprising the central repeat regions of the two major circulating vivax strains, VK210 and VK247, thus having the potential of providing worldwide coverage and afford protection against a large number of *P. vivax* strains [32]. Early assessment in mouse pre-clinical models indicated that the vaccine elicited antibodies able to recognize both, VK210 and VK247 vivax CSP molecules [33]. This vaccine has also been tested in Rhesus macaques. Despite this animal model cannot be challenged with *P. vivax* parasites, it can be useful to assess immune responses. Results upon vaccination of Rhesus macaques with VMP001 with GLA-SE adjuvant, a TLR4 agonist, were encouraging and all animals showed antigen-specific antibody responses as well as cellular immunity characterized by cytokine-producing CD4 cells [34]. Importantly, the vaccine has been assessed in a clinical trial, making use of a CHMI to determine vaccination efficacy against a sporozoite infection [8]. The trial consisted of a dose escalation in 30 volunteers, using three sequential immunisations of 15 μg, 30 μg, or 60 μg of the vaccine in AS01B adjuvant. The vaccine was safe and induced strong humoral and cellular immune responses. Importantly, a significant delay in time to parasitemia was observed and associated to antibody responses [8].

In summary, despite the tremendous disease burden caused by *P. vivax*, and the large number of people living in areas at risk of transmission, very few vivax vaccines have progressed to clinical trials. Low availability of funding to assess vivax vaccines may in part be responsible for the slow pace in the field. Similarly, the limited availability of tools to grow vivax in laboratory conditions causes very limited availability of the parasite, which has to be obtained from volunteers in endemic regions in order to be transported to institutions where an efficacy trial using CHMI can be performed. A CHMI is perhaps a basic need to identify suitable vaccine candidates with high efficacy levels, before planning field trials where high costs and difficult logistic measures require that highly efficacious vaccines progress to this stage. Ultimately, the efficacy in field trials of a vivax vaccine, if high, may renew the enthusiasm in the field to support development of more vaccines, or if low, may cause a continuous momentum of low funding to develop a *P. vivax* malaria vaccine.

## Declaration of Competing Interest

None.
